# The effect of perceived stress on organizational silence in emergency service doctors in Turkey: The mediating role of emotional intelligence

**DOI:** 10.3389/fpubh.2022.1010827

**Published:** 2022-10-26

**Authors:** Taskin Erdoğan, Yusuf Bayraktar, Fatih Uçan, Sait Sinan Atilgan

**Affiliations:** ^1^Faculty of Communication, Atatürk University, Erzurum, Turkey; ^2^Faculty of Tourism, Atatürk University, Erzurum, Turkey; ^3^Faculty of Economics and Administrative Sciences, Atatürk University, Erzurum, Turkey

**Keywords:** emergency service doctors, perceived stress, organizational silence, emotional intelligence, mediation effect, Hayes (2022) model 4

## Abstract

**Objectives:**

The purpose of this study is to determine the role of emotional intelligence in the relationship between the stress perceptions of emergency medicine doctors and their organizational silence behaviors.

**Methods:**

Data were collected digitally from 434 doctors working in emergency departments in Turkey. On the assumption that perceived stress was effective on organizational silence behavior and that emotional intelligence mediates this relationship, hypotheses were developed and a mediating effect model was established. The research model and hypotheses were shaped through Structural Equation Modeling (SEM). Hayes 4th model was used to test the research hypotheses. The research model was tested *via* SPSS Process v4.1 by Andrew F. Hayes.

**Results:**

According to the correlation analysis to determine the relationship between the variables, it was determined that perceived stress was positively correlated with organizational silence behavior, emotional intelligence was negatively correlated with perceived stress, and emotional intelligence was negatively correlated with organizational silence. As a result of the mediating effect model test, it was determined that emotional intelligence had a statistically significant mediating effect in the effect of perceived stress on organizational silence.

**Conclusion:**

Within the framework of the findings, it is thought that emotional intelligence is a key variable in turning the negative energy between stress and silence into positive.

## Introduction

Emergency Services Are one of the Most Important Parts of Health Services That Provide Uninterrupted Health Care for 7 Days and 24 Hours (1). Likewise, Emergency Services Are the Showcase of Health Institutions and the Patient Load Is Increasing day by day. In Addition, They Are Units Where Acute Cases Are Diagnosed and Treated Urgently (2). Apart From These, Emergency Services Are Expressed as the Units Where the Risks Are the Highest and Health Institutions Are the Most Chaotic (3). Due to all This Complexity, Risks and Intense Tempo, Emergency Service Workers Are under Intense Stress. It Has Been Reported That Emergency Service Workers Are Under High Stress Particularly due to the Reasons Such as Excessive Working Hours and Work Intensity of the Employees in Emergency Health Services, Working Above Their Capacity Most of the Time, Insufficient or no Rest Breaks, Insufficient Physical Conditions and Equipment, High Risks and Possibility of Occupational Accidents, High Probability of Verbal and Physical Violence, (3–5). Apart From all These Negativities, Patients and Their Relatives who Apply to the Emergency Services Are Also Very Stressful. This Further Increases the Stress of Healthcare Workers (2, 3).

Emergency Medical Services Are Teamwork and Involve Many Employees of Different Status, Expertise and Competence. In Addition to the Overcrowding and Workload of Emergency Departments, They Are Under More Stress Than Other Specialties due to the Fact That They Make a Decision and Start Treatment Immediately During Acute Illness (3). Due to the Stress They Are Exposed to, Emergency Service Workers may Give Different Reactions. These Reactions Negatively Affect the Physician's Relationship with Both the Patient/Patient Relatives and the Emergency Room Workers (6). Organizational Silence Behavior, Which Is one of These Negativities, Is the Individual's Staying Silent, Hiding or not Doing Things That He can Do or fix Voluntarily and Knowingly (7, 8).

Emotional Intelligence can be seen as an Important Tool for Minimizing the Negativities in Performance and Work Efficiency Caused by the Mental and Social Problems Experienced by Emergency Service Doctors Working Under Intense Stress (9). The Emergency Service Doctors can Empathize and Cope With the Negativities and Stress They Are Exposed to, by Emphasizing Emotional Intelligence and Providing Understanding, Interpretation and Analysis (10). Although Scientific Intelligence Is an Innate Hereditary Intelligence, Emotional Intelligence Is Seen as an Important Tool in Managing the Negative Effects of Stress Because It Is a non-Hereditary Type of Intelligence That can be Learned and Developed Over Time (11, 12). In Order to Provide Qualified and Comprehensive Health Services, Emotional Intelligence Should be Developed in Order to Cope With Emerging Stress Factors. Thus, It can be Thought That one of the Ways to Manage the Organizational Silence Triggered by Stress Is Through Emotional Intelligence. In the Literature, Studies on Stress, Silence and Emotional Intelligence of Healthcare Professionals, Particularly Emergency Physicians, Are Limited. The Results of This Research, Carried out in Order to Fill the gap in the Literature, Will Contribute to the Literature in Terms of Originality and Importance, Since They Represent all Emergency Service Doctors Working in Turkey. The Purpose of This Study Is to Investigate the Effect of Emotional Intelligence on Stress-Induced Silence Behavior in Emergency Service Doctors.

## Literature review

### Perceived stress and organizational silence

Stress is one of the Most Important Psychological Problems That Society and Individuals Often Face Throughout Their Lives. Emotional Tension Is Defined as Stress, Which Consists of the Interaction Between the Individual and His Environment and Mostly Disrupts the Psychological and Physiological Balance of the Individual (13, 14). Stress Is Discussed in a Broader Context as a Related Concept to Many Issues. Stress, Which Emerged as a Result of the Modern World, Has a Significant Impact on Our Lives and Is now Considered a Risk Factor for the Development of Various Diseases (15). In This Context, Perceived Stress Refers to a Series of Physical and Mental Tensions That Individuals Feel During Stimulating Events and Threats (16). It Is Also Associated With Increased Stress, Physical and Mental Health Problems (17).

Studies Highlighting the Negative Effects of Stress in Business Life Have Come to the Fore in Recent Years (18–22). However, Unlike Other Sectors, the Field of Health Prepares More Ground for the Formation of Stress. High Stress Levels Impose an Additional Burden on the Health Sector and Adversely Affect Public Health Outcomes in General (23). During the COVID-19 Pandemic, Frontline Medical Personnel Faced Tremendous Levels of Stress (24). AlDhaen (25) Supported With His Findings That the Unsuitable Working Environment Increases the Stress on Health Workers. Li et al. (26), in a Study They Conducted on Healthcare Professionals, They Stated That Doctors Experience Particularly High Risk and Stress During the COVID-19 Outbreak. This Study Found a Significant and Positive Relationship Between Depressive Symptoms and Organizational Silence. Chen et al. (27) Emphasized That Employee Silence Has Negative Effects on Organizations. Montgomery and Lainidi (28) in Their Study, Associated Employee Silence That Occurs in Health Services With Hiding Mistakes, Reducing Patient Safety and Covering up Mistakes Made by Others. Yang et al. (29), in Their Study With Nurses, It Was Suggested That Organizational Silence Represents an Important Problem for Health Institutions. It Has Been Stated That This Situation Will Negatively Affect the Development of Both Individuals and Organizations.

In the Literature, There Exist Studies That Have Established a Positive Relationship Between the Stress Arising From Working Life and Organizational Silence (30–33). It Is Seen That the Stress Arising in Business Life Is one of the Serious Problems Encountered in the Health Sector. In This Context, It Will be Possible to Reduce Stress by Making Some Arrangements Within the Organizational Structure (34). It Is Very Important for Doctors to be in Good Physical and Psychological Wellbeing in Order to Provide Better Service to Patients While Performing Their Duties. The Silence of Health Workers Within the Organization due to Stress can Have Harmful Long-Term Consequences for Emergency Services. It Is Thought That the Stress Arising Among Health Workers may Cause Organizational Silence. The Relationship Between Stress and Organizational Silence Has Been Revealed in the Literature, and Accordingly, Hypothesis 1 Has Been Developed to Determine the Level and Direction of This Relationship.

Hypothesis 1: There Is a Positive Relationship Between Perceived Stress and Organizational Silence.

### Perceived stress and emotional intelligence

Although Stress in Working Life Is not a Newly Emerged Concept, There Has Been a Significant Increase in Awareness About the Subject Recently. Today, Stress Is a Problem That Needs to be Overcome (35). Therefore, Stress Has Been Associated With Many Variables (24, 36–38). One of These Variables Is Emotional Intelligence. Emotional Intelligence Is Defined as a set of Interrelated Abilities That Allow Individuals to Properly Understand and use Emotional Information. Individuals With a High Ability to Understand the Feelings of Others can be More Sensitive to Their Thoughts and Feelings, as Well as Being Able to Perceive and Understand Others. Those who can Regulate and Manage Their Emotions can Direct Their Emotions to Constructive Results (39).

Studies That Establish a Direct Relationship Between Emotional Intelligence and Perceived Stress Are Found in the Literature. In These Studies, It Has Been Revealed That Emotional Intelligence Plays an Important Role in the Control of Emotions Arising From job Stress (40–43). Aghdasi et al. (44) Claimed a Negative Correlation Between Emotional Intelligence and Stress. Sy et al. (45) Revealed That Employees With High Emotional Intelligence Had low Occupational Stress. As a Result, They Stated That Individuals Could not Cope With Their Emotions in the Face of Difficult Conditions and Their job Satisfaction Was Negatively Affected due to too Much Stress. Nikolaou and Tsaousis (46) Found That There Was a Significant Negative Relationship Between Emotional Intelligence and job Stress. Bar-On (47) Claimed That Employees With High Emotional Intelligence Could Choose Better Coping Strategies Against Stress in Occupational Groups Where Stress Was Intense. DrSanthosh Kumar and Basha (48), in Their Study on Doctors, Suggested That Emotional Intelligence Factors Positively Affected Quality of Life. Another Study in the Field of Health by Landa et al. (49) Investigated the Relationship Between Emotional Intelligence and job Stress. The Results of Their Studies Showed That Nurses With High Emotional Clarity and Emotional Reparation Had Less Stress Levels, but Those With High Emotional Interest Had More Stress. The Relationship Between Perceived Stress and Emotional Intelligence Was Supported by the Literature, and Accordingly, Hypothesis 2 Was Developed to Determine the Level and Direction of This Relationship.

Hypothesis 2: There Is a Negative Relationship Between Perceived Stress and Emotional Intelligence.

### Organizational silence and emotional intelligence

Organizational Silence Constitutes an Important Obstacle in the Development of Organizations That Evaluate and Reflect the Differences Among Employees and Allow the Expression of Different Views (50). Emotional Intelligence, Which Contributes Positively to Cognitive-Based Performance and Means Using Different Abilities (51), can be Said to be an Important Factor in Whether Employees Engage in Silence Behavior or not.

Edwards et al. (52) Emphasized That Health Professionals may be Reluctant in Some Cases to Report Their Concerns, and That There Are Some Underlying Motives for Their Decision to Remain Silent. Srivastava et al. (53) Revealed That Employees With High Levels of Emotional Intelligence Could Evaluate and Control Their Emotions to Obtain Personal and Professional Benefits, and Could use Silence Effectively to Reduce the Negative Consequences of Working Life. Similarly, a Recent Study Claims That Emotional Intelligence Plays a key Role in Overcoming the Silence Caused by Management Weakness (54).

Taslak and Tunçel (55) Concluded That Employees' Emotional Intelligence Skills Had a Significant Effect on Organizational Silence Behaviors. Boadi et al. (56) and Ozen Kutanis et al. (57) Found a Negative Relationship Between Emotional Intelligence and Organizational Silence. These Studies Were Seen as Important in Terms of Revealing the Relationship Between Emotional Intelligence and Organizational Silence Behavior. In the Light of This Information, Hypothesis 3 Has Been Developed Since Emotional Intelligence Is Thought to be a key Position in Determining the Silence Behaviors of Employees.

Hypothesis 3: There Is a Negative Relationship Between Emotional Intelligence and Organizational Silence.

### Emotional intelligence as a mediator of the organizational silence-perceived stress relationship

Employees with high emotional intelligence are aware of their own emotions and can effectively control their emotions. This increases the likelihood of choosing strategies that will allow them to deal effectively with stressors (47, 58). In addition, it has been demonstrated by various studies in the literature that emotional intelligence plays a mediating role in tolerating negative situations that may arise in the organizational environment (59–63). For example, in a study conducted on resident doctors, it was emphasized that emotional intelligence plays a mediating role in the effect of perceived stress on burnout. It was determined that emotional intelligence had a mediating role in the effect of perceived stress on emotional exhaustion (64). Because employees with high emotional intelligence have the ability to monitor and analyze emotional interactions, they can better recognize cues about whether their supervisor is open to their voice or whether they prefer to keep their subordinates quiet (65). In this context, it is thought that emotionalv intelligence is an important factor between organizational silence and stress level. In the light of this information, it is important that emotional intelligence plays a mediating role in this study. Accordingly, hypothesis 4 was developed to test the mediating role of emotional intelligence in the relationship between perceived stress and organizational silence.

Hypothesis 4: Emotional intelligence has a mediating role in the effect of perceived stress on organizational silence.

## Methods

### Instruments

Perceived Stress Scale: The scale used was developed by Cohen et al. (66) and adapted into Turkish by Eskin et al. (67). Consisting of 14 items and 2 dimensions, PSS was developed to measure how individuals perceive stress. It was designed to measure perceptions of inadequacy (for example, in the past month, how often did you feel nervous and stressed?) and perceptions of discomfort (for example, how often did you feel over-whelmed in the past month?). Responses to different items are based on a Likert scale from 1 (never) to 5 (always).

Emotional Intelligence: The scale used is the Turkish version of the Emotional Intelligence Scale developed by Schutte *et al*. (68) and updated by Chan (69). This scale measures one's capacity to recognize and evaluate one's own emotions (e.g. I know why my emotions change), management and control of emotions (e.g. I hope good things will happen), empathetic sensitivity (e.g. I can understand people's emotions from facial expressions), and positive use of emotions (e.g. It consists of 12 items distributed among four factors that measure (I can solve problems better when I am in a positive mood). Responses to different items are based on a Likert scale from 1 (Strongly Disagree) to 5 (Strongly Agree).

Organizational Silence Scale: The scale used was developed by Dyne *et al*. (70). It was adapted in the studies of many researchers and finally it was adapted into Turkish by Çavuşoglu and Köse (71). There are six dimensions related to the concepts of voice and silence in the scale. This scale includes accepting silence (I am reluctant to talk about change proposals in our hospital on issues that are not relevant to me.), defensive silence (I do not suggest and say my ideas about change because I fear the reaction of the administrators in our hospital.), pro-social silence (I resist pressures on letting others know the information that can be considered secret for our hospital.), accepting voice (I act with the motivation of cooperation while expressing solutions to problems in our hospital, for the benefit of the institution.), defensive voice (I hesitate to support the ideas of my colleagues on issues that I am not related to in our hospital.), prosocial voice (I will focus the discussion on others to protect myself. It consists of five expressions and a total of 30 (thirty) expressions to measure each of the dimensions.

The reasons for using these scales in the study are that they have been used in many studies in Turkish culture, especially in the field of health (72–77). Confirmatory factor analysis (CFA) was performed to determine the validity of the scales within the scope of the study. The values of the fit indices confirm the scales (Table 1). These values are at a level that can be considered statistically reliable.

**Table 1:** Confirmatory factor analysis results of the scales used in the research.

**Scales**	***x*^2^/df**	**GFI**	**CFI**	**IFI**	**NFI**	**SRMR**	**RMSEA**	**Cronbach α**
Emotional intelligence	2.80	0.95	0.95	0.95	0.92	0.060	0.065	0.81
Organizational Silence	2.62	0.90	0.93	0.93	0.90	0.055	0.061	0.84
Perceived Stress	3.56	0.91	0.93	0.93	0.90	0.061	0.77	0.88

The values of the fit indexes confirm the scales. These values are at a level that can be considered statistically reliable. The fit indices displayed in Table 1 are considered adequate (78–83).

### Procedure

The study was approved by Atatürk University Institute of Social Sciences Research Ethics Committee and the officials of participating hospitals (Ref. SOSBIL 2021/2-7). Before the data were collected, they were presented to the expert team consisting of 4 experts and 4 academicians with experience in the field of emergency medicine for their evaluation of the scale items. The population of the study consists of about 4,000 emergency medicine physicians working in the field of emergency medicine in Turkey (84). A sample group consisting of 434 emergency medicine physicians was determined with the appropriate non-probabilistic sampling technique to represent the population. The data were collected by sending the link of the form created online *via* e-mail and WhatsApp. Data were collected over a three-month period from 17 December 2021 to 30 March 2022 from 434 emergency medicine physicians who agreed to participate in the study. All participants in the study were informed about the study and informed consent was obtained. Each questionnaire was filled in about 10 minutes. All doctors (without gender and age discrimination) working regularly in the emergency departments were included in the study.

### Data analysis

SPSS v25 was used to make descriptive statistics and correlation analyzes of the data within the scope of the study. SPSS Process v4.1 by Andrew F. Hayes was used to examine the mediating effect between emotional intelligence, perceived stress, and organizational silence. Confirmatory factor analyzes to test the validity of the scales were performed through the AMOS v20 program.

## Results

### Participants

The research was carried out with 434 doctors working in emergency departments in Turkey, aged between 21 and 36+ (sd= 2.61). Of the emergency service doctors participating in the study, 48.6% were women (*n* = 211) and 51.4% were men (*n* = 223). 40.3% (*n* = 175) of the doctors have a working time of less than 1 year, 27% (*n* = 117) of them have working years between 1 and 3 years, while 18.9% (*n* = 82) have 4–7 years and % 13.8 (*n* = 60) of them have 8 years or more working years.

### Correlation analysis

Correlation analysis was performed to show the mean standard deviation pairwise correlations between the variables. The results of the correlation analysis are shown in Table 2.

**Table 2:** Mean value, standard deviation and correlation of all variables (*n* = 434).

**Variable**	**M**	**SD**	**1**	**2**	**3**
1.Emotional Intelligence	3.897	0.461	1		
2.Organizational Silence	2.878	0.438	−0.244**	1	.
3. Perceived Stress	3.114	0.660	−0.420**	0.220**	1

*N* = 434, **p < 0.001.

The results showed that perceived stress has a positive relationship with organizational silence behavior (*r* = 0.220, *p* < 0.001), emotional intelligence has a negative relation-ship with perceived stress (*r* = −0.420, *p* < 0.001) and there is significant and negative relationship between emergency service doctors' emotional intelligence and organizational silence levels (*r*= −0.244, *p* < 0.001).

### Measurement model

This study, which aims to reveal the effects of stress levels and emotional intelligence levels on organizational silence of physicians working in the emergency department, was designed using quantitative research designs. In this direction, the research is based on the predictive relational screening design, which is considered among the non-experimental research designs by determining the relationships between the variables and estimating the effect levels (85).

### Structural model

**Figure 1:**
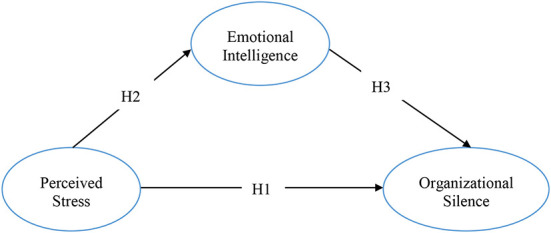
The mediating effect model.

A mediating effect model was used in this study, which aims to determine the role of emotional intelligence in the effect of perceived stress on organizational silence. The mediating effect model, which was created in order to test the hypotheses put forward in line with the purpose of the research, is shown in Figure 1. A mediating effect model was used in this study, which aims to determine the role of emotional intelligence in the effect of perceived stress on organizational silence. The mediating effect model, which was created in order to test the hypotheses put forward in line with the purpose of the research, is shown in Figure 1.

H1: There is positive a relationship between perceived stress and organizational silence level.
H2: There is a negative relationship between perceived stress and emotional intelligence.
H3: There is a negative relationship between emotional intelligence and organizational silence level.
H4: Emotional intelligence has a mediating role in the effect of perceived stress on organizational silence.

### The comparison test of mediating variables

**Figure 2:**
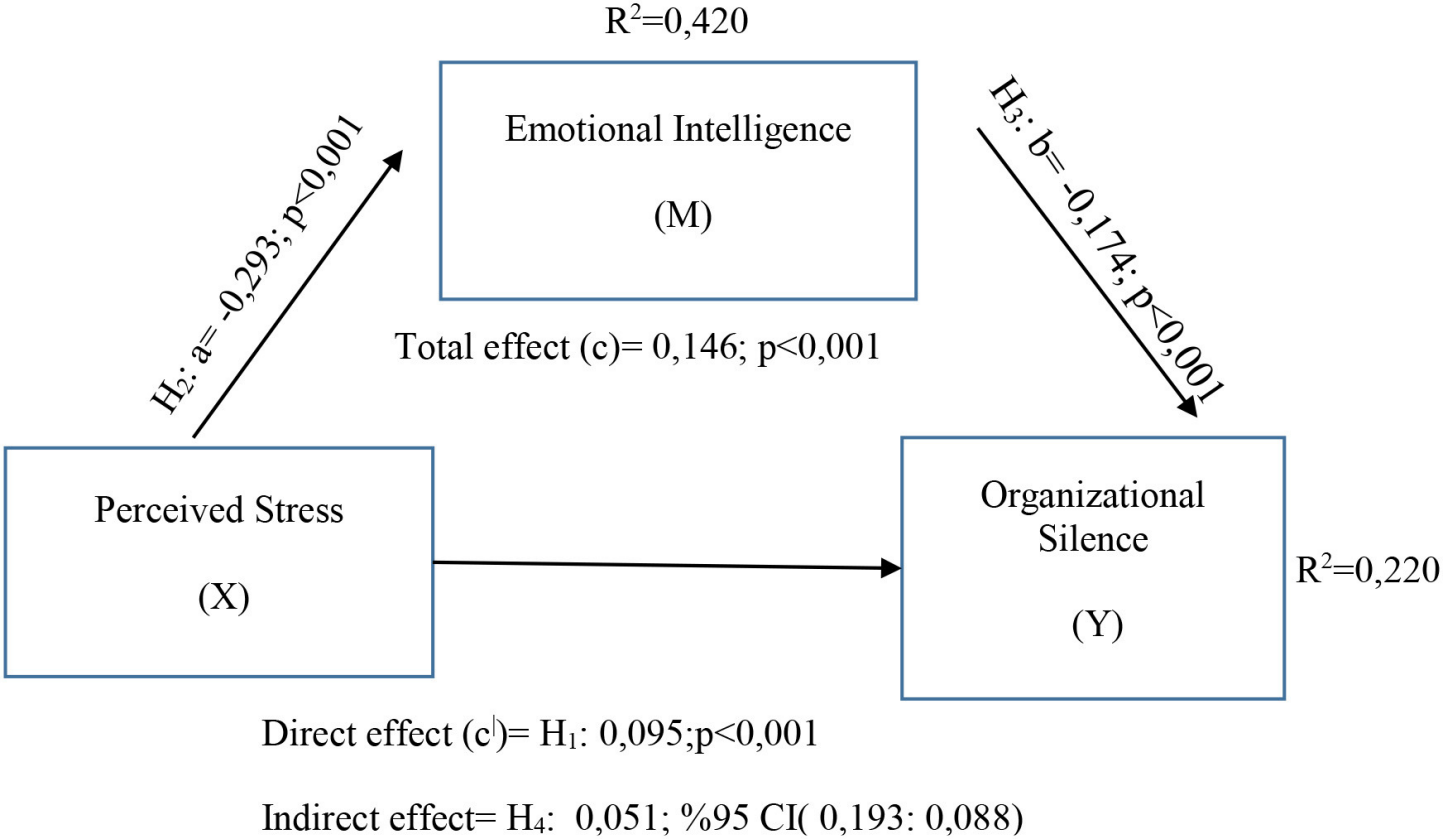
Mediator effect analysis.

The research was designed to examine the relationship between the variables of perceived stress, emotional intelligence and organizational silence. The research model and hypotheses were shaped through Structural Equation Modeling (SEM) in line with the purpose of the research. In order to test the research hypotheses, Hayes (86) 4th model was taken as a basis. The research model was tested *via* SPSS Process v4.1 by Andrew F. Hayes. Hypothesis and model test results are shown in Figure 2.

Process v4.1 by Andrew F. Hayes 4th Model created to test the research model is presented in Figure 2. Hypothesis tests were carried out in the light of the model created in line with Structural Equation Modeling (SEM), and the significance level of the relation-ship between the variables was examined with the Bootstrap technique at 95% confidence interval. Accordingly, the relationship between perceived stress and organizational silence (H1) was examined in the study. As seen in Figure 2, a significant (β = 0.095; *p* < 0.001; LLCI:0.0285; ULCI:0.1616) relationship was found between perceived stress and organizational silence. In line with this result, the hypothesis “H1: There is a relationship/causality between perceived stress and organizational silence level” was accepted. When the relationship (H2) between perceived stress and emotional intelligence is examined, it is seen that there is a significant and negative relationship (β = −0.293; *p* < 0.001; LLCI: −0.3535; ULCI: −0.2336). Accordingly, the hypothesis “H2: There is a relationship/causality between the perceived stress perception and emotional intelligence” was accepted. Research results support the relationship between emotional intelligence and organizational silence (H3) as significant and negative (β = −0.174; *p* < 0.001; LLCI: −0.2693; ULCI: −0.0789). In the light of this information, the hypothesis “H3: There is a relationship/causality between emotional intelligence and organizational silence level” was accepted.

The results of the mediating effect analysis performed to reveal the role of emotional intelligence (H4) in the effect of perceived stress on organizational silence are shown in Table 3. Accordingly, it is seen that emotional intelligence has a statistically significant mediating effect (β = 0.051; *R*^2^ = 0.220; LLCI: 0.0193; ULCI: 0.0088) in the effect of perceived stress on organizational silence. In this direction, it was determined that the hypothesis “H4: Emotional intelligence has a mediating role in the effect of perceived stress on organizational silence” was accepted. In line with the findings obtained in the research, the results of the hypothesis tests are summarized in Table 4.

**Table 3:** The role of emotional intelligence in the effect of perceived stress on organizational silence behavior (H4).

	**Result variables**			
	**Emotional intelligence organizational silence behavior**		**Emotional intelligence organizational silence behavior**	
	**β**	**SH**	**β**	**SH**
**Perceived stress (c** ^***i***^ **)**			0.095*	0.033
R^2^			0.220	
Perceived stress (a)	−0.293**	0.030		
R^2^	0.420			
Emotional Intelligence (b)			−0.174**	0.048
R^2^			0.220	
Total effect (c)			0.146	
Indirect Effect			0.051; %95 CI (0.193: 0.088)	

*p < 0.05, **p < 0.001.

**Table 4:** Hypothesis test results.

H1: There is positive a relationship between perceived stress and organizational silence level.	Accepted
H2: There is a negative relationship between perceived stress and emotional intelligence.	Accepted
H3: There is a negative relationship between emotional intelligence and organizational silence level.	Accepted
H4: Emotional intelligence has a mediating role in the effect of perceived stress on organizational silence.	Accepted

## Discussion

The aim of this study was to investigate the role of emotional intelligence in reducing organizational silence caused by perceived stress in emergency service doctors working in Turkey. Based on the data obtained from 434 employees working as emergency physicians in Turkey, it has been shown that emotional intelligence plays a mediating role in the relationship between perceived stress and organizational silence. Research makes theoretically little known phenomena more visible in practice. The difficulty of obtaining data for research from doctors working under intense work pressure such as emergency services and the fact that these data can represent the whole of Turkey make this study valuable. Mediation tests have been tried in the relationship between emotional intelligence and many variables, but no study has been found on how it mediates the delicate balance between stress and silence. In addition to these, as of the results of the study, emotional intelligence has come to the fore as an important actor in displaying silence behavior as a result of stress. These results contribute to our understanding of the psychological conditions that affect the mental health of emergency physicians. In addition, the study provides empirical evidence for the importance of using emotional intelligence for effective interventions in controlling stress-induced organizational silence in emergency service physicians. Based on this evidence, the present study has some theoretical and practical implications.

### Theoretical implications

This study proved that perceived stress level caused silence behavior in the work environment. It is one of the findings of this study that organizational silence behavior increases as the perceived stress level in emergency service doctors increases. It was found that emergency service doctors who reported higher levels of perceived stress exhibited more organizational silence behavior than others. Our findings are consistent with the results of similar studies in the health sector (26, 27). However, our findings are consistent with previous studies on teachers (33), white-collar workers (30), hotel workers (31), and heavy industry workers (32). Various studies have shown that emotional intelligence is a strong argument for tolerating negative situations that may arise in the organizational environment. Emotional intelligence, as in the health sector (42, 48, 87–92) have been the subject of research in many different fields (93–95). In these studies, it has been concluded that emotional intelligence plays a key role in solving problems in organizations and regulating emotions. The fact that emotional intelligence plays such an important role has formed the main motivation of our study and suggested that it may play a mediating role in the relationship between perceived stress and organizational silence. Therefore, the assumption that emotional intelligence has an effect on controlling the silence behavior caused by perceived stress has been the subject of this research. The findings obtained in this context confirm the hypothesis of the study. Accordingly, this research contributed to the literature with the conclusion that emotional intelligence plays a mediating role in the effect of perceived stress on organizational silence in emergency service doctors. Although the relationship between perceived stress and organizational silence has been discussed in the literature, the role of emotional intelligence in this relationship has not been mentioned. Emphasizing the importance of emotional intelligence as a method of coping with the stress-induced silence behavior, this research adds new information to the literature. This research has revealed empirical evidence that individuals' emotional intelligence plays an important role in eliminating stress-induced silence behavior. The research emphasizes that perceived stress not only directly affects organizational silence, but also has an indirect effect through emotional intelligence. Emphasizing the mediation of emotional intelligence in stress-induced silence behavior, this study provides important evidence to resolve the negative situation in this relationship. It is an important inference of this study that when emotional intelligence is higher, there is a decrease in silence behavior caused by stress.

### Practical implications

The findings of this study have a number of practical implications. Proposing that there is an ability-based model for emotional intelligence, Mayer et al. (96) highlighted that there was an intelligence aspect that could develop. Therefore, developing emotional intelligence in controlling the organizational silence behaviors of emergency physicians is an urgent issue that every healthcare institution and system should address.

There are studies showing that emotional intelligence is a skill that can be developed through education. In general, it has been emphasized that the high level of emotional intelligence is very important in solving the problems encountered (89, 97–99). Especially for doctors who have to work under high stress in the field of health, it will be possible to get positive results from activities to improve their emotional intelligence. In our study, this issue was brought to the fore and the important effect of emotional intelligence was revealed. Reshetnikov et al. (98) confirmed the effectiveness and importance of providing emotional intelligence training in health services and training highly qualified personnel. Knight et al. (100) suggested that it is necessary to develop emotional intelligence in the field of health. In a similar study, Cao et al. (99) presented specific and effective strategies for developing and guiding health professionals based on emotional intelligence in the health sector. While the results of these studies were an inspiration for our research, they also made us aware of the importance of focusing on the emotional intelligence of doctors working in emergency departments. We highlight the importance of using emotional intelligence to control silence in doctors working in emergency departments. Emotional intelligence training should be included in the education curricula of medical faculties and this aspect should be supported with artistic and social activities. Basically, managers should recognize the importance of emotional intelligence in controlling organizational silence behavior caused by perceived stress. In this context, managers should develop policies to keep and maintain communication channels within the organization.

## Conclusion

In conclusion, this study proved that emotional intelligence had an important role in controlling organizational silence caused by perceived stress in emergency service doctor. As it is known, emergency services are one of the most important units of the health sector and keeping the stress factor away from the employees is very important for the emergency services to stay more active. In addition, one of the places where employees' silence behavior is likely to have the worst consequences is again the emergency services. In this sense, it has been revealed that emotional intelligence, which emerges as a personal ability, has a feature that can eliminate these negativities. In this study, the importance of emotional intelligence in business life is repeated once again. Educating and equipping emergency service doctors with emotional intelligence has been seen as an important intervention style. Emotional intelligence acts as a psychological buffer in the stress-silence relationship.

## Limitation and future direction

The Findings of Our Study Should be Evaluated within the Framework of Some Limitations. First, in This Study, a Cross-Sectional Design Was Used, Which did not Allow Examining the Perceived Stress, Organizational Silence, and Emotional Intelligence of Emergency Service Doctors at Different Times. This Situation can be Considered as a Limitation in Interpreting the Findings Obtained From the Research Data. Future Research may Prefer a Longitudinal Design to Overcome This Limitation. Second, Since the Findings Were Obtained Only From Emergency Service Doctors, It Is Controversial That They can be Generalized to all Healthcare Professionals. Therefore, Future Studies may use Samples That Include Other Departments to Examine the Relationship Between Variables. Third, This Study Found That Emotional Intelligence Mediated the Relationship Between Perceived Stress and Organizational Silence. However, There may be Different Psychological Variables That Mediate This Relationship. Therefore, Possible Mediating Variables may Inspire Future Research. Fourth, the Emotional Intelligence Variable Examined in the Study Was Considered as a Single Dimension. However, Emotional Intelligence Consists of Different sub-Dimensions (One's Capacity to Recognize and Evaluate One's own Emotions, Management and Control of Emotions, Empathetic Sensitivity, Positive use of Emotions). The Fact That the sub-Dimensions of Emotional Intelligence Were not Taken Into Account in This Study can be Considered as a Limitation. In This Context, Future Research may Address the sub-Dimensions of Emotional Intelligence in the Stress-Silence Relationship. Finally, the Collection of Research Data Only From the Turkish Sample can be Expressed as a Limitation. Future Research can be Conducted in Different Countries to Reflect Economic, Cultural and Social Differences and These Findings can be Compared.

## Data availability statement

The raw data supporting the conclusions of this article will be made available by the authors, without undue reservation.

## Ethics statement

The studies involving human participants were reviewed and approved by Atatürk University Institute of Social Sciences Ethics Committee [Protocol Code: (Ref. SOSBIL 2021/2-7)]. The patients/participants provided their written informed consent to participate in this study.

## Author contributions

Conceptualization: TE, SA, and FU. Methodology: TE, SA, and YB. Software, visualization, surveillance, and note: YB and FU. Verification: YB and SA. Formal analysis and data curation: YB, SA, and FU. References: TE and FU. Writing original drafting, writing—review, editing, and project management: TE, YB, SA, and FU. All authors contributed to the article and approved the submitted version.

## Conflict of interest

The authors declare that the research was conducted in the absence of any commercial or financial relationships that could be construed as a potential conflict of interest.

## Publisher's note

All claims expressed in this article are solely those of the authors and do not necessarily represent those of their affiliated organizations, or those of the publisher, the editors and the reviewers. Any product that may be evaluated in this article, or claim that may be made by its manufacturer, is not guaranteed or endorsed by the publisher.
